# A Nutrigenetic Approach to Investigate the Relationship between Metabolic Traits and Vitamin D Status in an Asian Indian Population

**DOI:** 10.3390/nu12051357

**Published:** 2020-05-09

**Authors:** Buthaina E. Alathari, Dhanasekaran Bodhini, Ramamoorthy Jayashri, Nagarajan Lakshmipriya, Coimbatore Subramanian Shanthi Rani, Vasudevan Sudha, Julie A. Lovegrove, Ranjit Mohan Anjana, Viswanathan Mohan, Venkatesan Radha, Rajendra Pradeepa, Karani S. Vimaleswaran

**Affiliations:** 1 Hugh Sinclair Unit of Human Nutrition, Department of Food and Nutritional Sciences, University of Reading, Reading RG6 6AP, UK; B.E.A.A.Alathari@pgr.reading.ac.uk (B.E.A.); j.a.lovegrove@reading.ac.uk (J.A.L.); 2 Department of Food Science and Nutrition, Faculty of Health Sciences, The Public Authority for Applied Education and Training, PO Box 14281, AlFaiha 72853, Kuwait; 3 Department of Molecular Genetics, Madras Diabetes Research Foundation, Chennai 603103, India; bodhinid@gmail.com (D.B.); radharv@yahoo.co.in (V.R.); 4 Department of Biochemistry, Madras Diabetes Research Foundation, Chennai 600086, India; jayashri@drmohans.com; 5 Department of Foods, Nutrition and Dietetics Research, Madras Diabetes Research Foundation, Chennai 600086, India; lakshmipriya@mdrf.in (N.L.); s2r_7@mdrf.in (V.S.); 6 Department of Clinical Epidemiology, Madras Diabetes Research Foundation, Chennai 600086, India; kshan_rany@yahoo.com; 7 Department of Diabetology, Madras Diabetes Research Foundation & Dr. Mohan′s Diabetes Specialities Centre, WHO Collaborating Centre for Non-communicable Diseases Prevention and Control, ICMR Centre for Advanced Research on Diabetes, Gopalapuram, Chennai 600086, India; dranjana@drmohans.com (R.M.A.); drmohans@diabetes.ind.in (V.M.); guhapradeepa@gmail.com (R.P.)

**Keywords:** GRS, SNP, metabolic traits, vitamin D, 25(OH)D, carbohydrate intake, Asian Indian, CURES

## Abstract

Studies in Asian Indians have examined the association of metabolic traits with vitamin D status. However, findings have been quite inconsistent. Hence, we aimed to explore the relationship between metabolic traits and 25-hydroxyvitamin D [25(OH)D] concentrations. We investigate whether this relationship was modified by lifestyle factors using a nutrigenetic approach in 545 Asian Indians randomly selected from the Chennai Urban Rural Epidemiology Study (219 normal glucose tolerant individuals, 151 with pre-diabetes and 175 individuals with type 2 diabetes). A metabolic genetic risk score (GRS) was developed using five common metabolic disease-related genetic variants. There was a significant interaction between metabolic GRS and carbohydrate intake (energy%) on 25(OH)D (P_interaction_ = 0.047). Individuals consuming a low carbohydrate diet (≤62%) and those having lesser number of metabolic risk alleles (GRS ≤ 1) had significantly higher levels of 25(OH)D (*p* = 0.033). Conversely, individuals consuming a high carbohydrate diet despite having lesser number of risk alleles did not show a significant increase in 25(OH)D (*p* = 0.662). In summary, our findings show that individuals carrying a smaller number of metabolic risk alleles are likely to have higher 25(OH)D levels if they consume a low carbohydrate diet. These data support the current dietary carbohydrate recommendations of 50%–60% energy suggesting that reduced metabolic genetic risk increases 25(OH)D.

## 1. Introduction

Interaction between genetic and lifestyle factors have been shown to contribute to the development of metabolic disorders such as obesity and type 2 diabetes (T2D) [1,2]. The prevalence of metabolic diseases is increasing worldwide, and Asian Indians have a greater predisposition [3,4]. The Asian Indian population have a unique clinical phenotype characterized by increased visceral fat and waist circumference (WC), increased susceptibility to type 2 diabetes at a younger age, hyperinsulinemia, insulin resistance and dyslipidemia with raised triglycerides and low high density lipoprotein–cholesterol (HDL-c) levels at normal ranges of body mass index (BMI) collectively known as “Asian Indian Phenotype” [5,6]. Furthermore, several studies have demonstrated that metabolic diseases are associated with micronutrient deficiencies, such as vitamin D deficiency [7,8,9,10].

Vitamin D is a fat-soluble vitamin, known for its impact on skeletal and extra-skeletal physiological processes. Vitamin D deficiency exists in endemic proportions all over India, with a prevalence ranging from 80%–90% [11]. Adequate levels of vitamin D are important for calcium absorption, bone mineralization and skeletal growth as well as a multitude of biologic functions at the cellular level such as cell growth, proliferation, differentiation, inflammation and apoptosis. Additionally, vitamin D has been linked to cancer, cardiovascular diseases, inflammation and autoimmune diseases [12,13,14]. Several observational studies have associated vitamin D deficiency with increased obesity and reported inverse relationship between 25(OH)D concentration and BMI, WC and total body fat; however, the causal effect was not established [15,16]. Nevertheless, a Mendelian Randomization analysis in 42,024 participants of European ancestry concluded that increased BMI leads to reduced 25(OH)D concentrations while there was no causal association between lower 25(OH)D concentrations and higher BMI [17]. Given that observational studies are often prone to bias and confounding, a genetic approach to explain the relationship between metabolic diseases and vitamin D deficiency may be a better option to reduce any influence from unmeasured confounding factors.

Association of several genetic variants with metabolic diseases has been identified by candidate gene and genome-wide association studies (GWAS) [2,18,19,20]. Currently, the fat mass and obesity-associated (*FTO*) gene is the strongest risk loci for obesity [1,21]. The *FTO* gene is the first obesity susceptibility gene to be identified by two GWAS in European populations [22,23]. A study in an Asian Indian population has shown that lifestyle factors can influence the association of *FTO* gene with obesity traits [21]. Besides the *FTO*, Melanocortin 4 Receptor (*MC4R*) and Transcription Factor 7-Like 2 (*TCF7L2*) genes are the two commonly studied candidate genes for obesity and T2D [24,25,26,27,28,29,30,31,32,33,34,35,36,37,38]. In the present study, we examined the association of a metabolic-genetic risk score (GRS) developed from five single-nucleotide polymorphisms (SNPs) [*FTO* (rs8050136 and rs2388405), *MC4R* (rs17782313) and *TCF7L2* (rs12255372 and rs7903146)] with metabolic traits and vitamin D concentrations. In addition, we investigated the link between metabolic traits and vitamin D status by exploring the interactions between the metabolic GRS and lifestyle factors such as diet and physical activity on metabolic traits and vitamin D concentrations in an Asian Indian population.

## 2. Methods

### 2.1. Study Population

**Figure 1:**
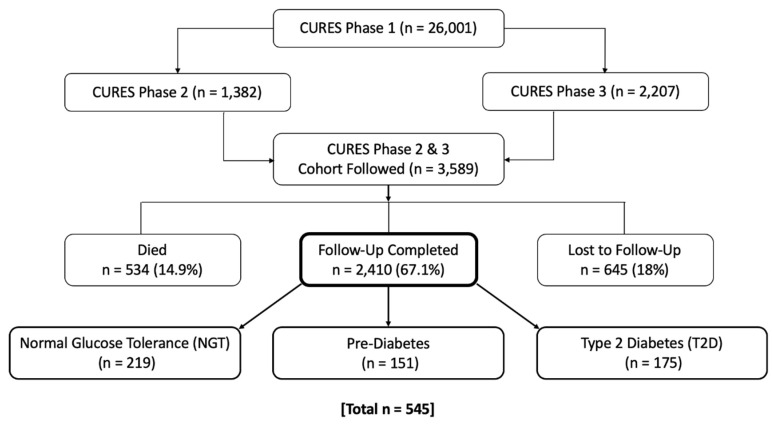
Selection of study participants from the Chennai Urban Rural Epidemiological Study (CURES follow-up study).

Five hundred forty-five study participants were recruited randomly from the Chennai Urban Rural Epidemiology Study (CURES) follow-up study (Figure 1), aged 29–85 years old [3]. CURES is an epidemiological cross-sectional study conducted on participants from Chennai city population in southern India, which is the fourth largest city in India. Details of the methodology have been previously published [39]. In brief, the CURES study was conducted in three phases. Phase 1: 26,001 adult participants (>20 years of age) were recruited using a systematic random sampling method covering the whole Chennai city and all participants were screened for diabetes. Phase 2: all 1382 diabetic participants were invited for further investigation (90.4% compliance). Phase 3: 2207 adult participants designated by way of every tenth participant from Phase 1, excluding diabetics, underwent further detailed investigations. Phases 2 & 3 constitutes the CURES follow-up cohort (*n* = 3589) [40]. For present study, 545 individuals were randomly selected from the follow-up cohort, which included: 219 normal glucose tolerant (NGT), 151 prediabetic and 175 T2D individuals. Three exclusion criteria were applied in this study: known cases of type 1 diabetes, diabetes secondary to other causes, and intake vitamin D supplements. The Madras Diabetes Research Foundation Institutional Ethics Committee granted ethical approval, and informed consent was obtained from the study participants. All clinical investigations were conducted according to the principles expressed in the Declaration of Helsinki (ICH GCP).

### 2.2. Anthropometric and Biochemical Measurements

Standardized methods were used to measure weight, height and WC. BMI was calculated based on the body weight in kilograms divided by the square of body height in meters. Generalized obesity was defined according to the World Health Organization Asia Pacific Guidelines for Asians (The Asia Pacific perspective 2000) as non-obese (BMI < 25 kg/m^2^) and obese (BMI ≥ 25 kg/m^2^) [41]. The following biochemical measurements were performed using kits supplied by Roche Diagnostics (Mannheim) on a Hitachi-912 Auto Analyzer (Hitachi, Mannheim, Germany): fasting plasma glucose (glucose oxidase-peroxidase), serum total cholesterol (cholesterol oxidase-phenol-4-amino-antipyrene peroxidase), serum triglycerides (glycerol phosphatase oxidase-phenol-4-amino-antipyrene peroxidase) and HDL-c (direct method; polyethylene glycol-pretreated enzymes) [42]. The Friedewald formula was used to calculate low-density lipoprotein cholesterol (LDL-c). Glycated hemoglobin (HbA1c) was determined by high-performance liquid chromatography using a Variant™ machine (Bio-Rad, Hercules, CA, USA). Serum insulin and 25(OH)D vitamin D concentrations were estimated using the electrochemiluminescence (ECLIA) using a Roche e601Cobas immunoassay analyzer (Roche Diagnostics, Indianapolis, IN, USA) [21]. The intra- and inter-assay coefficients of variation for vitamin D assay was 3.62% and 6.38%, respectively.

### 2.3. Assessment of Dietary Intake and Physical Activity

A validated, interviewer administered semi-quantitative food frequency questionnaire (FFQ) consisting of 222 different foods was used to assess dietary intake for the previous year [43]. In brief, participant had an interview ranging from 20 and 30 min where they had to estimate their usual portion size and usual frequencies (per day, week, month, year, never) with the help of visual aids of measurement equipment and food sizes. Description of the development of FFQ and the data on reproducibility and validity was previously published [43]. Daily average food and nutrient intake including macronutrient and total energy intake were analyzed and estimated by the EpiNu database system. A validated self-report questionnaire was used to assess physical activity levels of the participants [44]. Individuals were classified into three groups: 1. Vigorously active: where the participants exercised and engaged in demanding work activities; 2. Moderately active: where the participants either exercised or performed heavy physical work; 3. Sedentary: those participants who did not exercise or have physically demanding work.

### 2.4. SNP Selection and Genotyping

For the present study, five SNPs were chosen from three different genes based on their previous associations with obesity and T2D in several populations: *FTO* (rs8050136 & rs2388405) [21,25,26,38,45,46,47,48,49,50], *TCF7L2* (rs12255372 & rs7903146) [24,28,51,52,53,54,55] and *MC4R* (rs17782313) [25,27,34,37,38]. *FTO* gene variants are known to be the strongest genetic predictors of obesity to date [56,57]. The *FTO* SNP rs8050136 has shown a strong association with obesity and T2D [27,49,50,57,58]. Furthermore, the *FTO* SNPs, rs8050136 and rs2388405, have also been reported as intronic enhancers, as they may have an influence on the gene expression [45,59,60]. *MC4R* SNP rs17782313 was shown to be associated with obesity in European populations [34,38] and this finding then replicated in other populations including South Asians [27,37,61]. *TCF7L2* SNPs, rs12255372 and rs7903146, were shown to be associated with increased susceptibility to T2D in two large multiethnic meta-analyses [52,55]. Some studies have reported that the *TCF7L2* SNPs are involved in modulating and reducing adiposity through changes in the lifestyle [62,63,64]. Based on the previous studies, the above mentioned five SNPs were chosen for the present study.

Phenol–chloroform method of DNA extraction from whole blood was performed. The genotyping methodology for the five SNPs have been previously published [4,24,28]. Direct sequencing by an ABI 310 genetic analyzer (Applied Biosystems, Foster City, CA) was performed to confirm the efficiency of the genotyping; there was 99% concordance based on random duplicates of 20% of the samples.

### 2.5. Statistical Analysis

Statistical analyses were performed using SPSS statistical software (version 24; SPSS, Inc., Chicago, IL, USA). Allele frequencies were calculated by gene counting and chi-squared test was carried out to compare the proportions of genotypes/alleles. The genotypic frequencies of the five SNPs were in the Hardy–Weinberg Equilibrium (*p* > 0.05) (Table S1). To obtain normal distribution, all metabolic outcomes and vitamin D values were log transformed. The difference in the means of continuous variables between the participants with NGT vs. pre-diabetes and NGT vs. T2D was analyzed by independent sample t-test. Descriptive statistics for continuous variables are presented as means and standard deviation (SD). The chi-squared test was used to analyze and compare physical activity levels (vigorously active, moderately active and sedentary) between individuals with NGT vs. those with pre-diabetes and individuals with NGT vs. those with T2D. Unweighted metabolic GRS was calculated for each participant by adding the number of risk alleles for metabolic diseases. The SNPs, rs8050136, rs2388405, rs12255372, rs7903146 and rs17782313, were used to generate the GRS. A value of zero, one and two was assigned to each SNP, which indicates the number of metabolic disease-related risk alleles. These values were then calculated by adding the number of metabolic disease-related risk alleles across each SNP. The risk allele score was then divided by the median into those carrying ≤1 risk allele vs. those with >1 risk allele.

**Figure 2:**
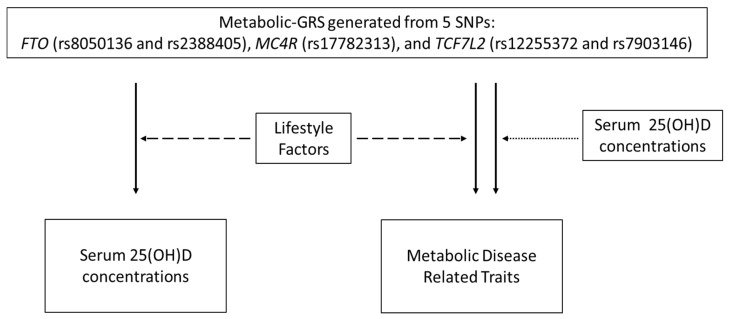
Study objectives. The unbroken one-sided arrows indicate the associations that were tested between the metabolic GRS and vitamin D concentrations and metabolic disease related traits. The broken one-sided arrows represent the interactions that were investigated between the GRS and lifestyle factors (diet and physical activity levels) on serum vitamin D and metabolic disease related traits. The one-sided dotted arrow indicates the interaction that was examined between metabolic GRS and 25(OH)D concentrations on metabolic disease -related traits.

A schematic representation of the study objectives is presented in Figure 2. Association analysis between the GRS and continuous and categorical variables were carried out using general linear and binary logistic regression models, respectively, adjusting for age, BMI, T2D and month of sample collection, wherever appropriate. The variable ‘month of sample collection’ was created based on the three seasons in India: summer (March to June), autumn/monsoon (July to October) and winter (November to February) [65]. Linear and logistic regression analyses were also used for investigating the interaction between SNPs and lifestyle factors (dietary intake and physical activity), where the interaction terms were incorporated into the models and adjusted for age, gender, BMI, T2D, total energy intake and month of sample collection wherever appropriate. Further tertile stratification of the lifestyle factor (diet/physical activity) was performed when there was a significant interaction between metabolic GRS and lifestyle factors on 25(OH)D concentrations and metabolic traits. Power calculation was not performed, given that there are no studies on metabolic GRS and no previously reported effect sizes for South Asians.

## 3. Results

### 3.1. Characteristics of Study Participants

The anthropometric, biochemical and lifestyle characteristics of the CURES participants are presented in Table 1. Significant differences were found between individuals with NGT, pre-diabetes and T2D, where individuals with T2D were older (*p* < 0.001), had higher WC (*p* < 0.001), fasting plasma insulin (*p* < 0.001), systolic and diastolic blood pressure (*p* < 0.001) and serum triglycerides (*p* < 0.001). However, individuals with pre-diabetes had higher BMI (*p* = 0.001) and LDL-c (*p* = 0.004) than individuals with NGT and T2D. No significant differences were observed in the levels of vitamin D, diastolic blood pressure, total cholesterol, HDL-c and dietary intakes across the three groups (*p* > 0.05).

### 3.2. Association of 25(OH)D Concentrations with Obesity and Type 2 Diabetes

There was a significant association of 25(OH)D concentrations with BMI (*p* = 0.017) and WC (*p* = 0.047) after adjusting for age, gender, month of sample collection and T2D. However, there was no association of 25(OH)D concentrations with fasting plasma glucose (*p* = 0.739), HbA1c (*p* = 0.823) and fasting plasma insulin (*p* = 0.387) after adjusting for age, gender, month of sample collection and BMI.

### 3.3. Association of the Metabolic GRS with 25(OH)D Level and Metabolic-Related Traits

No significant associations were observed between metabolic GRS and 25(OH)D concentrations (*p* = 0.34). None of the clinical and biochemical parameters such as BMI, WC, fasting plasma glucose and insulin, HbA1c, systolic and diastolic blood pressure, total cholesterol, HDL-c, LDL-c, and triglycerides, showed a significant association with metabolic GRS (*p* > 0.19 for all comparisons) (Table S2).

### 3.4. Interaction between Metabolic GRS and 25(OH)D Concentrations on Metabolic Traits

There was a borderline interaction between the metabolic GRS and 25(OH)D concentrations on HbA1c level (*p* = 0.048) after adjusting for age, gender, BMI, T2D and month of sample collection. However, no association was detected between the metabolic GRS and HbA1c when participants were grouped in tertiles of 25(OH)D concentrations (tertile 1, *p* = 0.471; tertile 2, *p* = 0.870; tertile 3, *p* = 0.486).

### 3.5. Interaction between Metabolic GRS and Lifestyle Factors on 25(OH)D Concentrations

**Figure 3:**
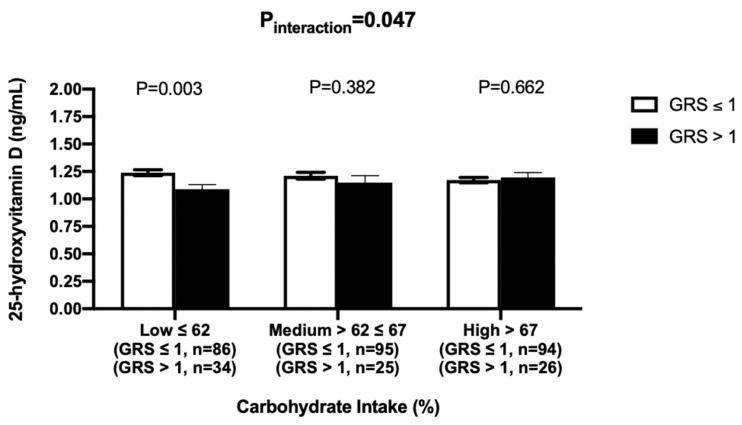
Interaction between metabolic GRS and log carbohydrate intake (%) on log 25 hydroxyvitamin D. White bars indicate individuals with GRS ≤ 1 risk allele; Black bars indicate individuals with GRS > 1 risk allele. Among individuals with low carbohydrates intake, those with < 1 risk allele had significantly higher 25 hydroxyvitamin D concentrations compared to those with > 1 risk allele (*p* = 0.003).

After adjusting for age, gender, BMI, T2D and month of sample collection, there was a significant interaction between the GRS and dietary carbohydrate intake on 25(OH)D concentrations (P_-interaction_ = 0.047). Tertile analysis was performed where individuals were grouped based on the tertiles of carbohydrate intake (energy%) [low ≤62%, medium = 62%–67% and high >67%)]. There were significant differences between the two GRS groups only among those who were in the first tertile of carbohydrate intake (*p* = 0.003), where individuals with lesser number of risk alleles (GRS ≤ 1) had greater 25(OH)D concentrations compared to those with higher number of risk alleles (GRS > 1) (Figure 3). Among individuals who had a higher carbohydrate intake (>67.28%), despite having lesser number of metabolic risk alleles, did not show a significant higher 25(OH)D concentrations (*p* = 0.66) compared to those with higher number of risk alleles (GRS > 1) (Figure 3).

### 3.6. Interaction between the GRS and Lifestyle Factors on Clinical and Biochemical Parameters Traits

**Figure 4:**
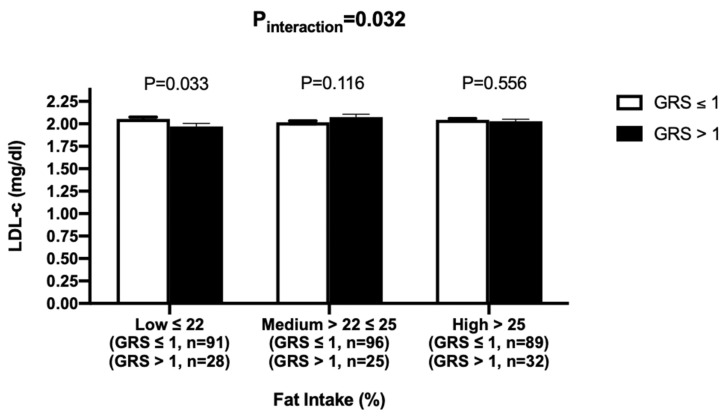
Interaction between metabolic GRS and fat intake (%) on log LDL-c. White bars indicate individuals with GRS ≤ 1 risk allele; Black bars indicate individuals with GRS > 1 risk allele. Among individuals consuming a low-fat diet, despite having a higher genetic risk (>1 risk allele), they had significantly lower LDL-c concentrations (*p* = 0.033).

There were significant interactions of metabolic GRS with dietary total fat intake and carbohydrates intake on LDL-c concentrations (P_interaction_ = 0.032 and P_interaction_ = 0.028, respectively) after adjusting for age, gender, BMI and T2D. However, no significant interaction was found between GRS and saturated, polyunsaturated and monounsaturated fat intake on LDL-c concentrations (*p* > 0.05, for all comparisons). After individuals were split into tertiles based on their fat intake (energy%) (low ≤22%, medium = 22%–25% and high >25%), there was a significant association of metabolic GRS with LDL-c in the low fat intake group (*p* = 0.033), where individuals despite having a higher genetic risk (>1 risk allele) had significantly lower LDL-c concentrations (Figure 4).

On the other hand, the tertile analysis of carbohydrate intake did not show any significant association between GRS and LDL-c concentrations (tertile 1, *p* = 0.453; tertile 2, *p* = 0.146; tertile 3, *p* = 0.460). None of the other lifestyle factors including physical activity showed a significant interaction with metabolic GRS on metabolic traits (*p* > 0.11 for all comparisons) (Table 2).

## 4. Discussion

Our study is the first to investigate gene-lifestyle interactions on 25(OH)D concentrations in Asian Indians. The main finding of our study is the interaction between carbohydrate intake and metabolic GRS, generated from five common metabolic-disease-related genetic variants, on 25(OH)D concentrations, where individuals who had less number of risk alleles (GRS ≤1) and consumed lower amounts of carbohydrates (≤62%) had significantly higher levels of 25(OH)D. Achieving and maintaining adequate levels of vitamin D is a desirable outcome as vitamin D deficiency is linked to several chronic diseases [66]. Given that previous studies have reported that Asian Indians have lower 25(OH)D concentrations [11,67], our findings suggest that, even if the genetic risk is lower, following the dietary carbohydrate recommendations (50%–60%) is required to improve the vitamin D status in this Asian Indian population.

Epidemiological studies have demonstrated a link between metabolic diseases such as obesity and T2D and vitamin D deficiency; however, it remains uncertain whether improving the metabolic status would reduce the risk of vitamin D deficiency [68,69,70,71]. The association between obesity and vitamin D status was consistent across different populations in several meta-analyses [70,71]. A large meta-analysis of 23 studies (*n* = 65,445) of mixed races reported that 35% of obese individuals suffer from vitamin D deficiency [70]. Several longitudinal studies have shown an inverse association between 25(OH)D status and T2D [66]. A meta-analysis in 2320 Caucasians showed that participants with adequate 25(OH)D concentrations had a 43% reduced risk of T2D [68]. However, unlike observational studies, vitamin D supplementation (4000 IU/day intake of vitamin D_3_) did not show beneficial effects on glycemic measures in two randomized, controlled trials (RCTs). In the first trial, well-controlled patients with T2D (*n* = 127) did not show any improvement in *β-*cell function, insulin secretion rate, nor in HbA1c levels after 48 weeks of supplementation [72]. In the second trial, 2423 prediabetic adults were evaluated for the development of diabetes for an average of 2.5 years; at the end of the study, 293 out of 1211 participants (24.2%) in the vitamin D supplementation group developed diabetes compared to 323 out of 1212 (26.7%) in the placebo group [73]. Furthermore, majority of RCTs also did not show an effect of vitamin D supplementation on weight loss [74].

Genetic studies are considered to be an effective approach in investigating the relationship between vitamin D and metabolic outcomes [75], as they are free from confounding and bias, which have been shown to affect the association between vitamin D levels and metabolic diseases. To our knowledge, there are no previous studies that have investigated the effect of metabolic disease-related genetic variants on vitamin D status. However, there are genetic association studies that have investigated the effect of vitamin D-related genetic variants on metabolic disease outcomes; but, the findings have been quite inconsistent [76,77,78,79]. A large bidirectional meta-analysis of 42,024 Europeans reported that there was no association between vitamin D-related genetic score and higher BMI; however, there was a significant association between genetically instrumented BMI and low vitamin D status [17]. In our study, the phenotypic associations of 25(OH)D concentrations with BMI and WC were statistically significant; but, the metabolic GRS did not show any association with 25(OH)D concentrations suggesting that the phenotypic associations are highly confounded.

To understand whether the genetic risk of metabolic diseases was influenced by vitamin D status, we tested for the interaction between the metabolic GRS and 25(OH)D concentrations on metabolic-disease related traits. None of the interactions were significant, except for a borderline interaction between the metabolic GRS and 25(OH)D concentrations on HbA1c level (*p* = 0.048); however, there was no association between metabolic GRS and HbA1c levels when participants were grouped into tertiles of 25(OH)D concentrations suggesting that there was no evidence for metabolic genetic risk acting as effect modifiers of the association between vitamin D status and metabolic traits. A study in 5160 participants of European ancestry also provided no evidence of vitamin D-related genetic variants acting as major modifiers of the association between 25(OH)D levels and cardio-metabolic risk [80]. Hence, these findings including the results from the present study indicate that vitamin D status is unlikely to have a significant impact on metabolic disease risk.

In the present study we found an interaction between the metabolic GRS and carbohydrate intake on vitamin D levels where lower consumption of carbohydrates was shown to be associated with higher 25(OH)D concentrations in the presence of reduced genetic risk. In the CURES, the carbohydrate intake included cereal grains, pulses, legumes, tubers, fruits, sweets, sweet beverages, carbonated beverages, junk food and added sugar, where consumption of refined cereals (i.e., mainly white rice) accounted for 78.1% of total calories [81]. This is a high intake compared to the recommended carbohydrate intake 50%–60% of total calories for Asian Indians [82], and the WHO (2002) recommendations of total carbohydrate intake at 55%–75% of total dietary energy [83]. The lowest tertile of the carbohydrate intake (≤62%), where we observed the positive association with vitamin D status, was close to the recommended dietary intake for Asian Indians (50%–60%), which supports the benefits of the current carbohydrate recommendations for Asian Indians. Our findings are also in line with a five-week intervention study [84], which used a reduced carbohydrate diet (43% carbohydrate; 27% fat) in 28 obese African American girls (9–14 years). The study showed that 25(OH)D concentrations were inversely associated with fasting glucose levels providing evidence that vitamin D may exert alterations in the biologic response to macronutrients such as dietary carbohydrates.

A further interesting finding in our study is the significant interaction between metabolic GRS and fat intake (%) on LDL-c concentrations, where despite having higher metabolic risk alleles, individuals who consumed a low-fat diet (≤21.89%) had significantly lower LDL-c levels. This suggests that lower dietary fat intake may influence the genetic risk of higher serum LDC-c concentrations, although mechanisms of action are unclear. This finding is in accordance with a GWAS on lipids in 541 individuals from the Quebec Family Study which reported an interaction between GRS (29 SNPs) and total fat intake on LDL-c concentrations (*p* << 10^−5^) [85]. The recommended dietary fat intake for Asian Indians is <30% [82]; however, in our study, only those individuals consuming total fat <21.8% demonstrated a significantly lower serum LDL-c concentrations, despite higher genetic susceptibility. Hence, our findings, if replicated using larger cohorts and dietary intervention studies, may have significant implications in providing dietary recommendations for those with higher metabolic-risk alleles.

The present study has several strengths, which include the use of a representative sample of Chennai [39] and an extensive and a validated semi-quantitative FFQ for dietary assessment [43]. In addition, the semi-quantitative FFQ has demonstrated high reproducibility and validity for macronutrient intakes such as dietary carbohydrate and fiber intake. Furthermore, the use of a metabolic GRS, which combines the effect of multiple SNPs, has been shown to increase the statistical power and an effective approach to study metabolic diseases [86,87]. However, there are some underlying limitations that need to be acknowledged. The measurement bias that is associated with self-reported FFQ and physical activity questionnaire cannot be ruled out. The study used a cross-sectional design and hence, no cause and effect conclusions can be established. Even though potential confounders were adjusted in all our statistical analyses, confounding factors such as sun exposure cannot be ruled out; however, we have adjusted for month of sample collection to overcome this limitation [65]. Finally, small sample size could be considered as another limitation in our study; nevertheless, we have identified significant findings, which suggest that the study is statistically powered to identify gene-diet interactions.

## 5. Conclusions

The present study has identified a novel interaction between metabolic GRS and carbohydrate intake on 25(OH)D levels in an Asian Indian population where individuals carrying a lesser number of metabolic risk alleles are likely to have higher 25(OH)D concentrations, only if they have a carbohydrate intake <62% energy. This is broadly in line with current dietary recommendations in India (50%–60% energy). This finding needs to be replicated in a larger cohort before these data can be confirmed. Mechanistic links also need to be identified.

## Figures and Tables

**Table 1:** Baseline characteristics of the study participants.

Characteristics of Study Participants	*n*	Normal Glucose Tolerance	*n*	Pre-Diabetes	*n*	Type 2 Diabetes	*p* Value
Age (years)	219	46.82 ± 10.54	151	47.79 ± 11.5	175	54.19 ± 11.04	<0.001 ^α γ^
BMI (kg/m^2^)	219	26.10 ± 5.15	151	27.95 ± 5.22	174	26.56 ± 4.58	0.001 ^β^
WC (cm)	219	86.04 ± 11.73	151	89.54 ± 11.2	173	90.11 ± 10.27	<0.001 ^α β^
Vitamin D (ng/mL)	219	19.55 ± 13.5	151	19.14 ± 10.47	175	17.8 ± 10.03	0.381
Fasting plasma glucose (mg/dL)	201	89.74 ± 6.54	144	103.43 ± 11.59	172	156.28 ± 64.43	<0.001 ^α β γ^
HbA1c (%)	219	5.61 ± 0.47	151	5.91 ± 0.59	175	8.19 ± 2.07	<0.001 ^α β γ^
Fasting plasma insulin (µLU/mL)	216	7.76 ± 5.13	139	8.13 ± 4.73	132	11.48 ± 7.69	<0.001 ^α γ^
Systolic BP (mmHg)	219	125.77 ± 20.97	151	126.54 ± 17.77	175	134.53 ± 19.6	<0.001^α γ^
Diastolic BP (mmHg)	219	79.17 ± 12.84	151	79.79 ± 10.78	175	80.67 ± 10.95	0.320
Total cholesterol (mg/dL)	219	181.07 ± 35.81	151	187.72 ± 35.28	175	181.2 ± 38.77	0.126
LDL cholesterol (mg/dL)	219	114.58 ± 31.58	151	119.17 ± 31.43	175	107.74 ± 34.63	0.004 ^α γ^
HDL cholesterol (mg/dL)	219	42.06 ± 9.57	151	40.10 ± 7.82	175	40.32 ± 8.58	0.093
Serum triglycerides (mg/dL)	219	122.15 ± 63.7	151	142.25 ± 83.08	175	165.71 ± 95.93	<0.001 ^α β γ^
Total energy intake (kcal)	185	2620.04 ± 752.02	83	2535.29 ± 803.78	93	2585.85 ± 787.79	0.609
Protein energy%	185	11.28 ± 1.19	83	11.31 ± 0.89	93	11.38 ± 1.2	0.758
Fat energy%	185	23.91 ± 4.76	83	23.33 ± 4.51	93	24 ± 4.72	0.582
Carbohydrate energy%	185	64.09 ± 6.69	83	64.89 ± 5.51	93	64.36 ± 5.97	0.556
Protein (g)	185	73.47 ± 21.39	83	71.59 ± 23.74	93	72.78 ± 21.2	0.704
Fat (g)	185	69.62 ± 25.15	83	65.92 ± 26.97	93	67.94 ± 22.15	0.407
Carbohydrate (g)	185	417.24 ± 115.73	83	409.91 ± 125.98	93	418.82 ± 142.37	0.847
Dietary fiber (g)	185	32.18 ± 10.91	83	30.77 ± 11.4	93	33.01 ± 11.85	0.235
Physical activity level	171	Sedentary (80.1%)	73	Sedentary (83.6%)	81	Sedentary (84.0%)	0.676 ^δ^
		Moderate (18.7%)		Moderate (13.7%)		Moderate (13.6%)	
		Vigorous (1.2%)		Vigorous (2.7%)		Vigorous (2.5%)	

Data shown are represented as means ± SD; *p* values were calculated using one-way ANOVA; ^δ^ *p* values were calculated using the chi-squared test. ^α^ indicates significance between non-diabetics and T2D individuals, ^β^ indicates significance between normal glucose tolerance and pre-diabetics, ^γ^ indicates significance between pre-diabetes and Type 2 diabetes. Abbreviations: CURES: Chennai Urban Rural Epidemiological Study, BMI: body mass index, WC: waist circumference, BP: blood pressure, LDL: low-density lipoprotein, HDL: high-density lipoprotein.

**Table 2:** Interaction between genetic risk score and lifestyle factors on clinical and biochemical parameters.

Outcome Measures	Physical Activity Levels	Protein%	Fat%	Carbohydrates%	Saturated Fatty Acids g/d *	Polyunsaturated Fatty Acids g/d *	Monounsaturated Fatty Acids g/d *
Body Mass Index	0.89	0.94	0.16	0.20	-	-	-
Waist Circumference	0.45	0.70	0.54	0.47	-	-	-
25(OH)D **	0.90	0.69	0.32	0.047	-	-	-
Fasting Plasma Glucose	0.12	0.90	0.16	0.09	-	-	-
Glycated Hemoglobin	0.13	0.52	0.44	0.32	-	-	-
Fasting Plasma Insulin	0.84	0.41	0.14	0.76			
Systolic Blood Pressure	0.72	0.19	0.96	0.62	-	-	-
Diastolic Blood Pressure	0.93	0.93	0.22	0.54	-	-	-
Total Cholesterol	0.80	0.40	0.47	0.55	-	-	-
Low density lipoprotein Cholesterol	0.90	0.12	0.032	0.028	0.21	0.28	0.27
High density lipoprotein cholesterol	0.68	0.55	0.72	0.80	-	-	-
Fasting serum triglycerides	0.87	0.11	0.26	0.11	-	-	-

*p* value for interactions obtained by general linear univariate analysis. All interactions were adjusted for age, gender, type 2 diabetes and BMI (except BMI) * Adjusted for log-total energy intake; ** Adjusted for month of sample collection.

## Supplementary Materials

The following are available online at https://www.mdpi.com/2072-6643/12/5/1357/s1, Table S1: Association Between Genetic Risk Score and Metabolic Traits, Table S2: Genotypic and allelic frequencies of the Single Nucleotide Polymorphisms that were used to create the genetic risk score.

## Abbreviations

T2D	type 2 diabetes
25(OH)D	25-hydroxyvitamin D
WC	waist circumference
BMI	body mass index
LDL-c	low density lipoprotein cholesterol
HDL-c	high density lipoprotein cholesterol
GWAS	genome wide association studies
*FTO*	fat mass and obesity-associated gene
*MC4R*	melanocortin 4 receptor gene
*TCF7L2*	transcription factor 7-like 2 gene
GRS	genetic risk score
SNP	single nucleotide polymorphism
CURES	Chennai Urban Rural Epidemiological Study
NGT	normal glucose tolerant
HbA1c	glycated hemoglobin
ECLIA	electrochemiluminescence
FFQ	food frequency questionnaire
SD	standard deviation
SBP	systolic blood pressure
DBP	diastolic blood pressure
