# Supplementary material for: A Nutrigenetic Approach to Investigate the Relationship between Metabolic Traits and Vitamin D Status in an Asian Indian Population
Source: Nutrients. 2020 May 9;12(5):1357. doi: 10.3390/nu12051357 (PMC7285077; doi:10.3390/nu12051357)
Supplement: Supplementary file 1 [file nutrients-12-01357-s001.pdf]

Table S1: Genotypic and allelic frequencies of the Single Nucleotide Polymorphisms that were used to create the genetic risk score

	Single Nucleotide Polymorphisms	Nucleotide Change	Homozygous Common	Heterozygote	Homozygous Rare	HWE P value	Minor Allele Frequency
FTO	rs8050136	A/C	CC: 290 (75.1%)	CA: 90 (23.3%)	AA: 6 (1.6%)	0.74	A= 0.13
	rs2388405	C/T	TT: 340 (83.5%)	CT: 62 (15.2%)	CC: 5 (1.2%)	0.26	C= 0.09
TCF7L2	rs12255372	G/T	TT: 163 (64.4%)	TG: 75 (29.6%)	GG: 15 (5.9%)	0.12	T= 0.21
	rs7903146	C/T	TT: 131 (51.8%)	TC: 98 (38.7%)	CC: 24 (9.5%)	0.37	T= 0.29
MC4R	rs17782313	C/T	TT: 144 (57.1%)	TC: 93 (36.9%)	CC: 15 (6.0%)	1.00	C= 0.24

HWE: Hardy Weinberg Equilibrium

Table S2: Association between genetic risk score (GRS) and Metabolic Traits

	GRS ≤ 1		GRS > 1		
	N	Mean ± SD***	N	Mean ± SD***	P value*
**BMI***	404	1.42 ± 0.08	141	1.43 ± 0.08	0.19
**WC**	403	1.94 ± 0.05	140	1.95 ± 0.06	0.53
**25(OH)D****	402	1.21 ± 0.25	141	1.19 ± 0.26	0.34
**Fasting plasma glucose**	380	2.04 ± 0.14	136	2.04 ± 0.13	0.32
**HbA1c**	403	0.80 ± 0.09	141	0.81 ± 0.108	0.96
**SBP**	403	2.10 ± 0.06	141	2.11 ± 0.07	0.23
**DBP**	403	1.90 ± 0.06	141	1.90 ± 0.06	0.37
**Fasting total cholesterol**	403	2.30 ± 0.09	141	2.24 ± 0.09	0.58
**Fasting LDL-c**	403	2.04 ± 0.15	141	2.02 ± 0.19	0.68
**Fasting HDL-c**	403	1.60 ± 0.09	141	1.60 ± 0.09	0.72
**Fasting serum triglycerides**	403	2.10 ± 0.22	141	2.10 ± 0.20	0.74

BMI: body mass index, WC: waist circumference, HbA1c: glycated Haemoglobin, SBP: systolic blood pressure, DBP diastolic blood pressure, LDL-c: low density lipoprotein, HDL-c: high density lipoprotein.

5 Single nucleotide polymorphisms: (*FTO* rs8050136 & rs2388405 + *TCF7L2* rs12255372 & rs7903146 + *MC4R* rs17782313)

All associations were adjusted for age, gender, type 2 diabetes and BMI
* Adjusted for age, gender and type 2 diabetes
** Adjusted for age, gender, type 2 diabetes, BMI and months of sample collection
*** log transformed values
